# Espiritualidade/Religiosidade e Adesão ao Tratamento em Indivíduos Hipertensos

**DOI:** 10.36660/abc.20240558

**Published:** 2025-02-04

**Authors:** Yanne da Silva Camargo, Aliny Serafim Borges Ferreira, Luana Araújo Macedo Scalia, Patrícia Magnabosco, Aline Guarato da Cunha Bragato, Maria Beatriz Guimarães Raponi, Nelson Dinamarco, Valéria Nasser Figueiredo

**Affiliations:** 1 Faculdade de Medicina Universidade Federal de Uberlândia Uberlândia MG Brasil Faculdade de Medicina da Universidade Federal de Uberlândia, Uberlândia, MG – Brasil; 2 Departamento de Clínica Médica Universidade Estadual de Santa Cruz Ilhéus BA Brasil Departamento de Clínica Médica, Universidade Estadual de Santa Cruz (UESC), Ilhéus, BA – Brasil

**Keywords:** Cooperação e Adesão ao Tratamento, Hipertensão, Adesão à Medicação, Espiritualidade

## Abstract

**Fundamento:**

A adesão ao tratamento medicamentoso e não medicamentoso para hipertensão tem um impacto socioeconômico importante, além de reduzir o risco de eventos cardiovasculares, morbidade e mortalidade. Sabe-se que a espiritualidade e a religiosidade podem ser incorporadas no manejo da hipertensão.

**Objetivo:**

Analisar possíveis fatores associados com a adesão ao tratamento em pacientes hipertensos e o papel da espiritualidade/religiosidade nesse contexto.

**Métodos:**

Estudo observacional, transversal, quantitativo, conduzido com 237 indivíduos hipertensos avaliados em um grande hospital de ensino no Brasil. Dados sociodemográficos, clínicos e de estilo de vida foram coletados, além das medidas antropométricas e realização do exame físico. Para determinar a adesão ao tratamento medicamentoso e não medicamentoso para hipertensão, utilizou-se o questionário QATSAH (Questionnaire of Adherence to the Treatment of Hypertension) e, para avaliar o nível de espiritualidade/religiosidade, foram aplicados o Inventário de Religiosidade de Duke e a Medida Multidimensional Breve de Religiosidade/Espiritualidade.

**Resultados:**

Níveis mais altos de adesão ao tratamento foram observados nos indivíduos com idade ≥65, fisicamente ativos, e que não consumiam bebida alcoólica (p<0,05). Em relação à religiosidade e à espiritualidade, a religiosidade intrínseca [β = 0,24, IC95% (0,22; 1,13), p = 0,004), valores e crenças (β = -0,18, IC95% [-1,58; -0,20]; p = 0,012), e perdão (β = 0,16; IC95% [0,13, 1,19], p = 0,015) foram preditores estatisticamente significativos de adesão ao tratamento. Religiosidade organizacional, religiosidade não organizacional, e experiências espirituais diárias não foram significativos.

**Conclusão:**

Religiosidade intrínseca mais alta, escores mais baixos de “valores e crenças” e escores mais altos de “perdão” aumentam o nível de adesão medicamentosa e não medicamentosa em indivíduos hipertensos.

## Graphical abstract

**Figura Central:**
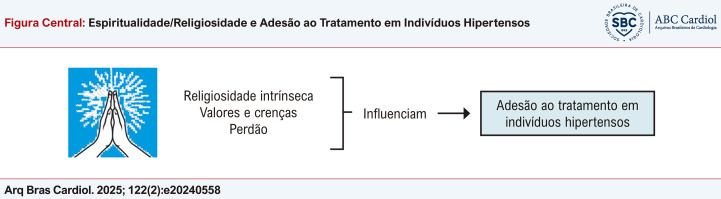
Espiritualidade/Religiosidade e Adesão ao Tratamento em Indivíduos Hipertensos

## Introdução

A hipertensão é uma doença multifatorial e multigênica, caracterizada por níveis elevados da pressão arterial (PA), que causam disfunção das artérias, e um dano progressivo e irreversível a órgãos-alvo. A hipertensão afeta 1,2 bilhões de pessoas em todo o mundo, levando à morte aproximadamente sete milhões de pessoas anualmente. Por se tratar de uma doença assintomática, a hipertensão está associada a um aumento na ocorrência de acidente vascular cerebral, insuficiência cardíaca congestiva, custos hospitalares, entre outros.^1,2^ Para o controle adequado da hipertensão, é essencial manter e controlar os níveis da PA. Tratamentos disponíveis incluem terapias farmacológicas e não farmacológicas; medidas não farmacológicas incluem perda de peso, mudanças na alimentação, restrições na ingestão de sódio e de álcool, e evitar o tabagismo.^3,4^

A adesão ao tratamento anti-hipertensivo é um processo multifatorial e complexo, por envolver a adesão do paciente a várias ações estabelecidas pelos profissionais de saúde para atingir um objetivo.^5^ No entanto, apesar da eficácia comprovada do tratamento da hipertensão, a adesão ainda é considerada baixa. A eficácia farmacológica pode variar entre 20 e 50%, e as taxas de adesão ao tratamento anti-hipertensivo são inferiores a 50% a partir de um ano após o diagnóstico de hipertensão.^6^ Pesquisadores observaram que menos de 14% dos indivíduos hipertensos têm a PA controlada, e essa porcentagem é inferior a 8% em países de renda baixa e média.^3^

Vários fatores contribuem para a adesão ao tratamento por pacientes hipertensos, incluindo a espiritualidade e a religiosidade,^7^ as quais auxiliam no convívio e no controle da doença. A religiosidade refere-se à conexão do indivíduo com o sagrado, geralmente expressa por crenças e práticas de uma religião específica ou de um grupo religioso. Por outro lado, a espiritualidade refere-se a um senso mais amplo de propósito e interconectividade, que podem ser vivenciados dentro ou fora do âmbito religioso.^7^ Pode-se inferir que indivíduos religiosos podem se beneficiar de conexões sociais mais fortes, o que encoraja a adoção de hábitos mais saudáveis e um controle do estresse mais eficaz. Isso pode levar a uma diminuição dos níveis de cortisol e melhor controle da PA. Práticas como oração e meditação promovem relaxamento, reduzindo a atividade do sistema nervoso simpático, o que, por sua vez, reduz a PA e a frequência cardíaca. Tais benefícios podem surgir da normalização do ritmo cardiovascular autonômico, de uma melhor modulação vagal, atividade simpática reduzida, melhor sensibilidade barorreflexa, e aumento da produção de óxido nítrico endógeno. Ainda, indivíduos religiosos assumem maior responsabilidade por sua saúde e bem-estar, e veem seus corpos como templos de Deus.^7,8^

Dado o aumento significativo na prevalência de hipertensão na população brasileira, aumentando de 22,6% em 2006 para 26,3% em 2023, além do alto número de mortes relacionadas a doenças hipertensivas (551 262 mortes entre 2010 e 2020),^9^ é crucial melhorar a adesão ao tratamento. Uma revisão sistemática avaliando a relação entre espiritualidade/religiosidade e a adesão medicamentosa em pacientes com doença cardiovascular relatou que uma compreensão mais profunda dessa conexão pode auxiliar no desenvolvimento de estratégias para aumentar a adesão ao tratamento, que sejam culturalmente sensíveis, baseadas na espiritualidade, e centradas no paciente. Com base nesses achados, nosso objetivo é identificar aspectos específicos da espiritualidade e da religiosidade que influenciem a adesão ao tratamento, abordando uma lacuna na literatura em que muitos estudos reconhecem a importância potencial dessas dimensões, mas não exploram elementos práticos.^10,11^

## Objetivos

O objetivo do presente estudo foi analisar os fatores potencialmente associados com a adesão ao tratamento em pacientes hipertensos e o papel da espiritualidade e da religiosidade nesse contexto.

## Métodos

Este é um estudo exploratório, transversal, descritivo, com abordagem quantitativa, conduzido nos departamentos de emergência, clínica médica e cirurgia de um grande hospital universitário. O hospital é um centro referência para atendimento de alta e média complexidade para municípios localizados no Triângulo Mineiro e Alto Paranaíba pelo Sistema Único de Saúde (SUS).

A amostra incluiu 237 pacientes hipertensos admitidos no hospital mencionado. Para o cálculo do tamanho amostral, foi obtido o número dos pacientes hipertensos admitidos no hospital em 2021 (n=4126). Uma amostra representativa foi determinada com um nível de confiança de 95% e uma margem de erro de 5%, resultando em um tamanho amostral de 237 indivíduos. Utilizou-se a fórmula de Cochran, assumindo-se uma proporção populacional de 50%,^12^ um intervalo de confiança de 95%, e uma margem de erro de 5%.

A fórmula é a seguinte: $N=z^{2}\cdot p(1-p)/E^{2}$ onde:

*• z* é o valor z correspondente ao intervalo de confiança desejado;
*• p* é a proporção esperada do atributo na população; e
*• E* é a margem de erro

O pesquisador selecionou os pacientes a partir de uma lista de internações diárias, incluindo somente os pacientes com números pares, até alcançar o tamanho amostral desejado.

Foram incluídos pacientes internados, com idade superior a 18 anos, de ambos os sexos, que possuíam um diagnóstico de hipertensão e haviam recebido tratamento medicamentoso por pelo menos seis meses, e tinham um escore “Mini-Mental” maior que 25 pontos.^13^ Gestantes e indivíduos com doenças psiquiátricas graves ou deficiências mentais (caracterizadas por sintomas como confusão, delírio, fala ou comportamento desorganizado, alucinações, e alterações extremas de humor) foram excluídos.

A coleta de dados foi realizada entre dezembro de 2021 e junho de 2022. Foi usado um questionário preparado pelos próprios pesquisadores, composto de dados sociodemográficos (sexo, idade, raça autorrelatada, escolaridade, estado civil, renda, religião, profissão), dados clínicos (peso, altura, PA, frequência cardíaca e pulso), claudicação intermitente, comorbidades (diabetes, obesidade, estilo de vida, tabagismo, consumo de álcool) e medicamentos em uso (anticoagulantes, agentes antiplaquetários, Inibidores de Enzima Conversora de Angiotensina – IECA, Bloqueadores do Receptor de Angiotensina – BRA, Bloqueadores de Canal de Cálcio – BCC, betabloqueadores, estatinas, nitrato e diuréticos).

Para a medida do peso corporal, utilizou-se uma escala digital eletrônica do tipo plataforma, com capacidade para 150 Kg e sensibilidade de 50 g (Omron HBF-214). A altura foi medida com auxílio de uma fita métrica inelástica fixada em uma parede, com os participantes de pé com as costas contra a parede. O Índice de Massa Corporal (IMC) foi calculado pela fórmula peso (Kg) / altura (m)^2^, e os valores considerados normais (IMC entre 18,5 e 24,9 Kg/m^2^), sobrepeso (IMC entre 25 e 29,9 Kg/m^2^) e obesidade (IMC ≥30 Kg/m^2^).^14^

Para a medida da PA, foram utilizados aparelhos portáteis (modelo HEM-7113 Omron) e manguitos adequados para circunferência do braço. As medidas da PA foram tomadas seguindo-se as diretrizes brasileiras de hipertensão arterial,^1^ com os participantes sentados, manguito posicionado ao nível do coração, e a palma da mão virada para cima. Durante a medição, os participantes ficavam com as pernas descruzadas, bexiga vazia, e eram orientados a não praticarem exercício físico por pelo menos 60 minutos antes do exame, e a não ingerirem bebida alcoólica, café ou alimentos, nem fumarem dentro de 30 minutos antes da medição. Todos os participantes foram submetidos a três medições da PA, com intervalo de um minuto entre as medidas, e a média das duas últimas medidas da PA foi considerada para análise.

Indivíduos diabéticos foram definidos pelo uso de hipoglicemiantes e/ou insulina, de acordo com dados obtidos dos prontuários médicos, antes da internação hospitalar. Em relação à dislipidemia, foram considerados os pacientes que apresentavam alterações em pelo menos um dos componentes (HDL-C, LDL-C ou triglicerídeos), e pacientes utilizando estatinas. Pacientes que fumavam um ou mais cigarros por dia foram considerados fumantes. Pacientes que não praticavam nenhuma atividade física por pelo menos dez minutos contínuos durante a semana foram considerados sedentários. Quanto ao consumo de bebidas alcoólicas, todos os pacientes que relataram o consumo foram considerados para análise, independentemente do tipo, da quantidade e da frequência da bebida.

Para avaliar a adesão ao tratamento medicamentoso e não medicamentoso, após obtenção da autorização do autor, foi usado o *Questionnaire of Adherence to the Treatment of Hypertension* (QATSAH), que consistia em 12 perguntas, com um coeficiente alfa de Cronbach de 0,81. A escala pode variar entre 60 e 110, e quanto maior o escore, maior a adesão ao tratamento (Nível 60: Os pacientes não tomam a medicação anti-hipertensiva pelo menos uma vez por semana ou tomam a medicação na dose diferente da prescrita pelo menos uma vez por semana; Nível 70: Os pacientes não tomam a medicação anti-hipertensiva no horário correto pelo menos uma vez por semana e comparecem a consultas agendadas; Nível 80: Os pacientes não tomam a o medicamento anti-hipertensivo na dose prescrita pelo menos uma vez por mês; os pacientes tomam a medicação independentemente de sentir ou não sintomas, e reduzem o consumo de sal e gordura em um terço; Nível 90: Os pacientes não tomam a medicação no horário correto pelo menos uma vez por mês, e reduzem pela metade o consumo de sal, gordura e doces e bebidas doces, tomam o medicamento pelo menos uma vez ao ano e praticamente não ingerem gordura, doces ou bebidas doces; Nível 100: Os pacientes não tomam a medicação anti-hipertensiva pelo menos uma vez ao ano e praticamente não ingerem gordura, doces ou bebidas doces; Nível 110: Os pacientes nunca deixam de tomar a medicação anti-hipertensiva, praticamente não ingerem sal e seguem o tratamento não medicamentoso rotineiramente.^15^

Para avaliar a influência da espiritualidade/religiosidade, foi utilizada a escala DUKE (P-durel), composta por cinco itens que medem três dimensões do envolvimento religioso relacionado a desfechos de saúde: Religiosidade Organizacional (RO), Religiosidade Não-Organizacional (RNO) e Religiosidade Intrínseca (RI).^16^ A RO envolve participação em serviços religiosos e atividades comunitárias associadas à religião, enquanto RNO refere-se a práticas privadas não religiosas tais como oração, meditação, e ações pessoais de devoção. Por outro lado, a RI foca na importância e na influência da religião na vida diária do indivíduo.^16^

Ainda, foi utilizada o *Brief Multidimensional Measure of Religiousness/Spirituality* (BMMRS-p) para avaliar a influência da religiosidade/espiritualidade sobre a adesão ao tratamento. O instrumento avalia a relação entre religiosidade, espiritualidade e saúde. O BMMRS-p possui 38 itens e mede 11 dimensões: 1) Experiências espirituais diárias; 2) Valores/crenças; 3) Perdão; 4) Práticas religiosas privadas; 5) Ações religiosas de enfrentamento; 6) Apoio religioso; 7) História religiosa espiritual; 8) Comprometimento; 9) RO; 10) Preferências religiosas; e 11) Autoavaliação global de espiritualidade/religiosidade. As opções de respostas estão organizadas em uma escala do tipo Likert, com alguns itens variando de uma a oito opções, e outras de uma a seis opções. Cada dimensão possui um sistema específico de pontuação, em que um escore mais baixo indica um grau mais alto da dimensão em questão. Para facilitar a análise, os escores podem ser revertidos durante a inserção dos dados, de modo que escores mais altos refletem uma maior religiosidade/espiritualidade.^17^

### Análise estatística

Os dados foram organizados em uma planilha Excel, validados por digitação dupla, e a análise estatística foi realizada usando o programa *Statistical Package for the Social Science* (SPSS Windows), versão 25.0^TM^. As variáveis contínuas foram descritas por médias ± desvios padrões devido à normalidade da distribuição dos dados, e as variáveis categóricas foram expressas usando frequências relativas e absolutas. O teste de Kolmogorov-Smirnov foi usado para avaliar a normalidade da distribuição dos dados. O teste t de Student não pareado foi usado para avaliar a influência das variáveis sociodemográficas e clínicas, bem como dos hábitos de vida, sobre os escores de adesão ao tratamento. A influência simultânea das variáveis foi analisada usando regressão linear múltipla, em que as variáveis relacionadas à religião e à espiritualidade foram inicialmente incluídas juntas no modelo. A multicolinearidade foi avaliada usando o Fator de Inflação da Variância (FIV) e o diagnóstico de colinearidade, e não foram identificadas questões significativas. Com base na importância conceitual e significância estatística, um modelo final com nove variáveis preditoras foi estabelecido. As presunções para testes paramétricos foram verificadas, e um nível de significância de 5% (α = 0,05) foi adotado para análises inferenciais. Como apresentado na Tabela 4, o poder estatístico alcançado foi 98,7%, considerando o tamanho amostral de 237 participantes, nível de significância de 0,05, nove variáveis preditoras, e um coeficiente de determinação R^2^ = 0,12.

**Tabela 4:** – Análise de regressão linear múltipla dos fatores sociodemográficos e religiosos/espirituais que influenciam a adesão medicamentosa e não medicamentosa em pacientes hipertensos, com base no *Questionnaire of Adherence to the Treatment of Hypertension* (QATSAH); Uberlândia, Minas Gerais, Brasil, 2021-2022

Variáveis	Β	Beta	Sig.	IC 95%
Sexo	-0,47	-0,03	0,63	[-2,39,1,45]
Idade	1,89	0,12	0,073	[-0,18,3,96]
Renda	1,93	0,12	0,059	[-0,07,3,93]
Educação	0,52	0,03	0,63	[-1,63,2,68]
Comorbidades	0,15	0,03	0,56	[-0,36,0,66]
Religiosidade intrínseca	0,67	0,24	0,004	[0,22,1,13]
Experiências espirituais intrínsecas	0,09	0,06	0,46	[-0,16,0,35]
Valores/crenças	-0,89	-0,18	0,012	[-1,58,-0,20]
Perdão	0,66	0,16	0,015	[0,13,1,19]

*QATSAH: Questionnaire of Adherence to the Treatment of Hypertension; Variáveis dicotômicas: sexo (masculino ou feminino – categoria de referência); idade (adulto ou idoso – categoria de referência); renda (< R$2500,00 e > R$2500,00 – categoria de referência); educação (ensino fundamental e ensino médio incompleto – categoria de referência).*

## Resultados

Foram incluídos 237 pacientes com idade média de 63,0±12,1 anos. A maioria dos pacientes era do sexo masculino (127; 53,6%), com idade superior a 60 anos (156; 65,8%), católicos (134; 56,5%), com mais de oito anos de educação formal (166; 70%), aposentados (133; 56,1%), e com sobrepeso (156; 67,8%); 44,7% (n=106) eram brancos, e 46,8% (n=111) dos pacientes apresentaram alto nível (percentil 90) de adesão ao tratamento anti-hipertensivo. A Tabela 1 apresenta o perfil sociodemográfico e clínico dos pacientes hipertensos, bem como hábitos de vida, medicamentos usados, e níveis de adesão de acordo com o questionário QATSAH.

**Tabela 1:** – Perfil clínico e sociodemográfico, hábitos de vida, medicamentos, e níveis de adesão de acordo com o questionário QATSAH nos participantes hipertensos (n=237); Uberlândia, Minas Gerais, Brasil, 2021-2022

Variáveis	Valores
Homens, n (%)	127 (53,6%)
Idade, anos	63,03±12,06
≥65 anos, n (%)	156 (65,9%)
Raça branca, n (%)	106 (44,8%)
Educação formal	227 (95,8%)
Casados, n (%)	104 (41,2%)
Renda < 1SM, n (%)	145(63,4%)
Religião	
• Católica, n (%)	134 (56,4%)
• Evangélica, n (%)	57 (24%)
• Espírita, n (%)	17 (7,2%)
• Sem religião, n (%)	23 (9,7%)
• Não acredita em Deus, n (%)	1 (0,4%)
• Candomblé, n (%)	5 (2,1%)
Aposentado, n (%)	133 (56,2%)
IMC, kg/m^2^	28,29±6,27
PAS, mmHg	128,86±21,91
PAD, mmHg	75,91±13,09
Frequência cardíaca, n (%)	76,90±14,69
Pulso regular, n (%)	211 (88,9%)
Claudicação intermitente	58 (24,5%)
DM, n (%)	78 (32,8%)
Obesidade, n (%)	42 (17,7%)
Dislipidemia, n (%)	91 (38,4%)
IM, n (%)	57 (24%)
AVC, n (%)	29 (12,2%)
ICC, n (%)	49 (20,6%)
Sedentarismo, n (%)	154 (65,1%)
Tabagismo, n (%)	32 (13,6%)
Consumo de bebida alcoólica, n (%)	36 (15%)
Uso de medicamentos	
• Anticoagulantes, n (%)	16 (6,8%)
• Agentes antiplaquetários, n (%)	25 (10,7%)
• Inibidores de ECA, n (%)	29 (12,3%)
• BRA, n (%)	144 (61,3%)
• BCC, n (%)	44 (18,7%)
• BB, n (%)	88 (37,6%)
• Estatina, n (%)	75 (31,9%)
• Nitrato, n (%)	8 (3,5%)
• Diurético, n (%)	116 (49,4%)
QATSAH	
• Nível 60	1 (0,42%)
• Nível 70	11 (4,6%)
• Nível 80	80 (33,7%)
• Nível 90	111 (46,8%)
• Nível 100	31 (13,0%)
• Nível 110	3 (1,26%)

*Valore expressos em frequências absolutas (n) e relativas (%) ou em média e desvio padrão; SM: salário mínimo; IMC: índice de massa corporal; PAS: pressão arterial sistólica; PAD: pressão arterial diastólica; DM: Diabetes Mellitus; IM: infarto do miocárdio; AVC: acidente vascular cerebral; ICC: insuficiência cardíaca congestiva; ECA: enzima conversora de angiotensina; BRA: bloqueadores do receptor de angiotensina; BCC: bloqueadores do canal de cálcio; BB: betabloqueadores; QATSAH: Questionnaire of Adherence to the Treatment of Hypertension.*

Observou-se um nível mais alto de adesão ao tratamento nos indivíduos com idade ≥ 65 anos, não sedentários, e que não ingeriam bebida alcoólica (p<0,05) (Tabela 2).

**Tabela 2:** – Comparação entre variáveis clínicas, sociodemográficas, hábitos de vida e média obtida no *Questionnaire of Adherence to the Treatment of Hypertension* (QATSAH); Uberlândia, Minas Gerais, Brasil, 2021-2022

	Média - QATHAS	p
Sexo feminino	91,8 ±8,3	0,620
Sexo masculino	92,3 ±7,2	
Idade ≥65 anos	93 ±7,9	0,009*
Idade <65 anos	90,4 ±6,9	
Brancos	91,8 ±7,6	0,567
Mistos (morenos)/Negros/ Asiáticos	92,4 ±7,8	
Educação não formal	88,3 ±5,3	0,444
Educação formal	92,3 ±7,7	
<1 SM	91,9 ±7,9	0,476
≥1 SM	92,6 ±7,4	
Não sedentário	94,9 ±7,6	0,000*
Sedentário	90,6 ±7,3	
Fumantes	91,6 ±6,7	0,689
Não fumantes	92,7 ±7,8	
Não consumiam bebidas alcoólicas	92,5 ±7,9	0,031*
Consumiam bebidas alcoólicas	90 ±5,8	

*SM: salário mínimo; teste t de Student.*

Uma análise de regressão linear múltipla foi realizada para avaliar a influência de fatores religiosos e espirituais sobre a adesão (Tabela 3). Religiosidade intrínseca, valores e crenças, e perdão foram significativamente associados com resultados mais altos no QATSAH. Escores mais altos na religiosidade e perdão se correlacionaram com maior adesão ao tratamento anti-hipertensivo, enquanto escores mais baixos nos valores e crenças se associaram com melhor adesão (Figura Central).

**Tabela 3:** – Análise de regressão linear múltipla dos fatores religiosos e espirituais que influenciam a adesão medicamentosa e não medicamentosa em pacientes hipertensos, com base no *Questionnaire of Adherence to the Treatment of Hypertension* (QATSAH); Uberlândia, Minas Gerais, Brasil, 2021-2022

Variáveis religiosas e espirituais	Β	Beta	Sig.	IC 95%
Religiosidade organizacional	0,364	0,081	0,308	[-0,34,1,07]
Religiosidade não organizacional	0,158	0,026	0,735	[-0,76,1,08]
Religiosidade intrínseca	0,586	0,208	0,023	[0,08,1,09]
Experiências espirituais intrínsecas	0,122	0,081	0,380	[-0,15,0,39]
Valores/crenças	-1,061	-0,219	0,002	[-1,74,-0,38]
Perdão	0,696	0,170	0,012	[0,16,1,23]
Ações religiosas de enfrentamento	-0,187	-0,088	0,283	[-0,53,1,04]
Comprometimento	-0,042	-0,041	0,613	[-0,20,0,12]
Autoavaliação global de espiritualidade/religiosidade	0,367	0,083	0,287	[-0,30,0,15]

Com base nessa análise mais ampla, quatro variáveis chaves – religiosidade intrínseca, experiências espirituais diárias, valores e crenças, e perdão – foram selecionadas para ajuste devido à importância referencial e significância estatística. Essas variáveis foram então ajustadas quanto aos fatores sociodemográficos (sexo, idade, renda, educação e comorbidades), como mostrado na Tabela 4. No modelo ajustado, religiosidade intrínseca, valores e crenças, e perdão continuaram significativos, reforçando ainda mais sua influência sobre adesão.

## Discussão

O envelhecimento está geralmente associado com uma incidência mais alta de hipertensão, uma condição que requer intervenções farmacológicas para controlar a PA. Em muitos casos, idosos tendem a ter mais consciência da importância do cuidado à saúde, que inclui adesão regular à medicação prescrita. Neste estudo, observou-se que indivíduos com idade igual ou superior a 65 anos apresentaram níveis mais altos de adesão ao tratamento. Esse fato pode ser justificado pelo conhecimento sobre a importância do tratamento para prevenir complicações, visita regular a consultas médicas, e estabilidade na rotina diária, com horários mais regulares de refeições e medicamentos entre a população mais idosa.^18,19^

A relação positiva entre ser fisicamente mais ativo e a adesão ao tratamento nos pacientes hipertensos destaca-se como um fator importante na manutenção da saúde cardiovascular. Além da associação positiva entre controle da PA e atividade física regular, sabe-se que a atividade física pode reduzir valores da PA até 24 horas após a prática.^20^ Além disso, indivíduos que incorporam atividade física na rotina diária tendem a aderir a prescrições médicas e a outras diretrizes relacionadas a um estilo de vida saudável, incluindo manutenção do peso corporal, controle do estresse e sono de qualidade. Esses dados corroboram os resultados deste estudo, em que os participantes não sedentários apresentaram níveis mais altos de adesão ao tratamento.

Existe um consenso entre os estudos que o consumo excessivo de álcool pode exercer uma influência negativa na PA, aumentando os riscos de hipertensão e comprometendo a eficácia dos medicamentos anti-hipertensivos, e constituindo uma importante barreira à adesão ao tratamento. Neste estudo, observou-se que os participantes que não consumiram álcool apresentaram níveis mais altos de adesão ao tratamento. Indivíduos que optam por não consumir álcool geralmente demonstram um maior comprometimento às recomendações médicas, que podem incluir o uso regular das medicações prescritas. Ainda, a não ingestão de bebida alcoólica contribui para a manutenção de um peso corporal adequado, reduzindo o risco de obesidade, o que pode melhorar o quadro clínico da hipertensão.^21,22^

A religiosidade e a espiritualidade foram identificadas em vários estudos como fatores que promovem a adesão ao tratamento medicamentoso. Por exemplo, um estudo encontrou que indivíduos com níveis mais altos de religiosidade são mais propensos a adotar estilos de vida mais saudáveis, o que pode contribuir para uma maior adesão ao tratamento.^23^ Ainda, estudos indicam que pacientes que realizam práticas como oração e meditação, ou buscam apoio em suas comunidades religiosas tendem a apresentar menos sintomas de depressão, o que pode facilitar a adesão ao tratamento.^23^ Vários mecanismos podem explicar esse fenômeno. A religiosidade e a espiritualidade podem fomentar um senso de esperança e propósito, o que aumenta o comprometimento do indivíduo ao tratamento. Ainda, o envolvimento da comunidade e algumas práticas como evitar certos alimentos e substâncias prejudiciais, em geral encorajadas por grupos religiosos, também podem exercer um papel chave na promoção de adesão.^23,24^

A religiosidade intrínseca refere-se à experiência profunda e pessoal de fé, em que a espiritualidade exerce um papel significativo na vida do indivíduo. Os dados listados aqui indicam que quanto maior a religiosidade intrínseca de um paciente hipertenso, maior a adesão ao tratamento. Um estudo conduzido com 9581 mulheres e homens adultos adventistas relatou que a religiosidade intrínseca é um fator significativo na redução das taxas de hipertensão.^25^ A razão para essa associação ainda não está clara, mas podemos pensar que indivíduos com alta religiosidade intrínseca encontram na sua fé uma fonte de apoio emocional, força interior e sentido para enfrentar os desafios, incluindo o cuidado com a saúde. Assim, a crença em um propósito maior pode motivar a adoção de comportamentos saudáveis e a adesão a recomendações médicas, contribuindo, assim, a um melhor controle da hipertensão. Uma religiosidade intrínseca elevada pode ser um recurso valioso na promoção da adesão ao tratamento da hipertensão, ao prover suporte emocional, um senso de propósito e ao influenciar positivamente hábitos de vida saudáveis. No entanto, é essencial considerar a individualidade de cada pessoa ao explorar essa interconexão entre espiritualidade e saúde.^26^

Neste estudo, o perdão também esteve relacionado a uma maior adesão ao tratamento. Entende-se “perdão” como a capacidade de se livrar de ressentimento e hostilidade em relação aos outros. Indivíduos que cultivam um desejo de perdoar geralmente vivenciam níveis menores de estresse, ansiedade e raiva, os quais, quando presentes em excesso, podem contribuir para o desenvolvimento e a piora da hipertensão.^27^ Em um estudo conduzido com 3105 pacientes hipertensos, encontrou-se que a variável perdão associou-se com valores menores de PA diastólica e uma menor probabilidade de desfechos hipertensivos.^28^ Indivíduos submetidos a um programa de treinamento de perdão tiveram reduções significativas na PA em comparação àqueles que não participaram do programa.^29^ Portanto, podemos inferir que a capacidade de perdoar está associada a uma melhora na saúde psicológica, promovendo um estado de bem-estar que pode se refletir positivamente nas escolhas de estilo de vida e na adesão a recomendações médicas.

O perdão também está relacionado à promoção de comportamentos saudáveis. Indivíduos que cultivam a capacidade de perdoar podem ser mais propensos a adotar uma abordagem proativa no cuidado da sua saúde, incluindo uma adesão consistente à medicação, alimentação equilibrada e atividade física regular. Esses elementos são fundamentais no controle eficaz da hipertensão. Além disso, a prática do perdão está associada a uma melhoria na relação interpessoal e social. Relações familiares e comunitárias mais saudáveis podem criar um ambiente de apoio em que a adesão ao tratamento é encorajada e facilitada. O apoio social é reconhecido como um fator crucial no manejo efetivo da hipertensão e na promoção de comportamentos saudáveis.^28,30^

Contudo, em relação a valores e crenças, observou-se uma influência negativa sobre a adesão ao tratamento. Valores e crenças referem-se às convicções fundamentais sobre como as pessoas interpretam e interagem com o mundo, ou seja, eles são elementos intrínsecos da identidade de cada indivíduo, formatando suas percepções sobre saúde, tratamento e estilo de vida. Assim, quando os valores e as crenças vão de encontro às recomendações médicas para o controle da hipertensão, a adesão ao tratamento pode ser comprometida. Crenças culturais, espirituais e filosóficas podem exercer um papel significativo em como uma pessoa aborda o tratamento da hipertensão. Ainda, uma desconfiança em relação à eficácia de tratamentos convencionais, em geral baseada em crenças pessoais, pode levar à busca de abordagens alternativas ou hesitação em seguir as recomendações médicas.^31^

Portanto, identificar e apoiar ações religiosas de enfrentamento e estratégias de perdão, bem como compreender os valores e as crenças dos pacientes hipertensos, podem promover e facilitar maior adesão ao tratamento. É crucial que profissionais da saúde exerça um papel ativo nesse processo, não só reconhecendo e validando a importância da espiritualidade e das práticas religiosas para seus pacientes, como também integrando esse entendimento às estratégias de cuidado personalizado. Fazendo isso, os profissionais podem identificar possíveis barreiras à adesão ao tratamento baseadas em crenças e atuar de maneira colaborativa para encontrar soluções que respeite essas visões enquanto encorajam práticas efetivas de saúde.^32^

### Limitações do estudo

Devido ao delineamento transversal, tamanho amostral limitado, e o fato de o estudo ter sido conduzido em um único hospital, inferências causais e a generalização dos resultados a outros contextos devem ser feitas com cuidado. Além disso, a natureza transversal do estudo limita a capacidade de se estabelecer uma relação de causa e efeito entre os fatores, como espiritualidade/religiosidade e adesão ao tratamento nos pacientes hipertensos. Ainda, a maioria dos dados foram autorrelatados, o que introduz um potencial viés de memória. A adesão foi avaliada indiretamente usando autorrelatos dos pacientes por meio do questionário QATSAH.

## Conclusão

A religiosidade/espiritualidade influenciou a adesão medicamentosa e não medicamentosa em indivíduos hipertensos, nas dimensões “religiosidade intrínseca”, “valores e crenças” e “perdão”. Esses achados sugerem que investigações e intervenções focadas em elementos psicossociais e comportamentais são essenciais para melhorar os desfechos de saúde da população em termos do controle/manutenção da hipertensão.
