# Supplementary material for: Espiritualidade/Religiosidade e Adesão ao Tratamento em Indivíduos Hipertensos
Source: Arq Bras Cardiol. 2025 Feb 4;122(2):e20240558. [Article in Portuguese] doi: 10.36660/abc.20240558 (PMC11870022; doi:10.36660/abc.20240558)
Supplement: Translation

# Translation: Spirituality/Religiosity and Adherence to Treatment in Hypertensive Individuals

**DOI:** 10.36660/abc.20240558i

**Authors:** Yanne da Silva Camargo, Aliny Serafim Borges Ferreira, Luana Araújo Macedo Scalia, Patrícia Magnabosco, Aline Guarato da Cunha Bragato, Maria Beatriz Guimarães Raponi, Nelson Dinamarco, Valéria Nasser Figueiredo

**Affiliations:** 1 Brazil Faculdade de Medicina da Universidade Federal de Uberlândia, Uberlândia, MG – Brazil; 2 Brazil Departamento de Clínica Médica, Universidade Estadual de Santa Cruz (UESC), Ilhéus, BA – Brazil

**Keywords:** Treatment Adherence and Compliance, Hypertension, Medication Adherence, Spirituality

## Abstract

**Background:**

Adherence to drug and non-drug treatment for hypertension has a major socioeconomic impact, in addition to reducing the risk of cardiovascular events and morbidity and mortality. It is known that spirituality and religiosity can be incorporated into coping and managing hypertension.

**Objective:**

To analyze possible factors associated with adherence to treatment in hypertensive patients and the role of spirituality/religiosity in this context.

**Methods:**

Observational, cross-sectional, quantitative study, carried out with 237 hypertensive individuals monitored in a large Brazilian teaching hospital. Sociodemographic, clinical and lifestyle data were collected, in addition to measuring anthropometric data and performing a physical examination. To determine adherence to drug and non-drug treatment for hypertension, the QATSAH instrument was used and, to assess the level of spirituality/religiosity, the Duke Religion Index and the Brief Multidimensional Measure of Religiousness/Spirituality were applied.

**Results:**

Higher levels of adherence to treatment were observed when aged ≥65 years, physically active, and who did not consume alcohol (p<0.05). Regarding religiosity and spirituality, intrinsic religiosity (β = 0.24, 95%CI [0.22, 1.13], p = 0.004), values and beliefs (β = -0.18, 95%CI [-1.58, -0.20], p = 0.012), and forgiveness (β = 0.16, 95%CI [0.13, 1.19], p = 0.015) were statistically significant predictors of treatment adherence. Organizational religiosity, non-organizational religiosity, and daily spiritual experiences were not significant.

**Conclusion:**

Greater intrinsic religiosity, lower scores in Values and Beliefs and higher scores in Forgiveness increase the level of medication and non-medication adherence in hypertensive individuals.

## Introduction

Hypertension consists of a chronic multifactorial and multigenic disease, characterized by high blood pressure (BP) levels, which causes impairment of the arteries, and leads to progressive and irreversible damage to target organs. Hypertension affects 1.2 billion people around the world and leads to the death of approximately seven million individuals annually. As it is an asymptomatic disease, it is associated with an increase in stroke, congestive heart failure, hospital costs, among others.^1,2^ In order to control hypertension properly, it is essential to maintain and control BP levels. Among available treatments are pharmacological and non-pharmacological strategies; non-pharmacological measures include weight reduction, dietary changes, sodium restriction, alcohol restriction and discouraging smoking.^3,4^

Adherence to hypertension treatment is a multifactorial and complex process, as it involves compliance by the hypertensive individual with several actions established by the health professional to achieve a goal.^5^ However, despite proving the effectiveness of hypertension treatment, adherence is still considered low. Pharmacological effectiveness can vary between 20 and 50%, and adherence rates to pharmacological antihypertensive treatment are below 50% from one year after diagnosis of hypertension onwards.^6^Researchers observed that less than 14% of hypertensive individuals have their BP controlled, and this percentage is below 8% in low- and middle-income countries.^3^

Several factors contribute to treatment adherence in hypertensive patients, including spirituality and religiosity,^7^ which have been shown to aid in coping with and managing hypertension. Religiosity refers to an individual’s connection with the sacred, often expressed through the beliefs and practices of a specific religion or religious group. Spirituality, on the other hand, relates to a broader sense of purpose and interconnectedness, which can be experienced within or outside a religious framework.^7^ It can be inferred that religious individuals may benefit from stronger social connections, which encourage the adoption of healthier habits and more effective stress management. This can lead to reduced cortisol levels and improved BP control. Practices like prayer and meditation promote relaxation, reducing sympathetic nervous system activity, which in turn lowers BP and heart rate. These benefits may arise from the normalization of autonomic cardiovascular rhythms, enhanced vagal modulation, decreased sympathetic activity, improved baroreflex sensitivity, and increased production of endogenous nitric oxide. Additionally, religious individuals often take greater personal responsibility for their health and well-being, viewing their bodies as temples of God.^7,8^

Given the significant rise in the prevalence of hypertension among the Brazilian population, increasing from 22.6% in 2006 to 26.3% in 2023, along with the high number of deaths related to hypertensive diseases (551,262 deaths between 2010 and 2020),^9^ the improvement of treatment adherence is crucial. A systematic review examining the relationship between spirituality/religiosity and medication adherence in patients with cardiovascular disease found that a deeper understanding of this connection can support the development of culturally sensitive, spiritually-based, and patient-centered strategies to enhance medication adherence. Building on these findings, our goal is to identify specific aspects of spirituality and religiosity that influence treatment adherence, addressing a gap in the literature where many studies recognize the potential importance of these dimensions but do not explore actionable elements.^10,11^

## Objectives

The objective of the present study was to analyze the factors potentially associated with treatment adherence in hypertensive patients and the role of spirituality/religiosity in this context.

## Methods

This is an exploratory, cross-sectional, descriptive study with a quantitative approach, conducted in the departments of emergency medicine, internal medicine and surgery of a large Brazilian university hospital. The hospital serves as a referral center for medium- and high-complexity care for municipalities located in the Triângulo Mineiro and Alto Paranaíba regions through the Unified Health System (SUS).

The sample included 237 hypertensive patients who were admitted to the aforementioned hospital. To calculate the sample size, the number of hypertensive patients admitted to the hospital in 2021 (n = 4,126) was obtained. A representative sample was determined with a 95% confidence level and a 5% margin of error, resulting in an estimated sample size of 237 subjects. Cochran’s formula was used, assuming a population proportion of 50%,^12^ a 95% confidence level, and a 5% margin of error. The formula is as follows: $N=z^{2}\cdot p(1-p)/E^{2}$ where:

*• z* is the z-value corresponding to the desired confidence level,
*• p* is the expected proportion of the attribute in the population, and
*• E* is the margin of error

The researcher selected patients from the daily hospitalization list, including only those with even numbers, until the desired sample size was reached.

Included participants were over 18 years old, had a clinical diagnosis of hypertension, were of both sexes, were hospitalized, had been undergoing medication treatment for at least six months, and had a Mini-Mental score greater than 25 points.^13^ Pregnant women and individuals with severe psychiatric illnesses or mental disabilities (characterized by symptoms such as confusion, delusions, disorganized speech or behavior, hallucinations, and extreme mood swings) were excluded.

Data collection took place between December 2021 and June 2022. A questionnaire, prepared by the researchers themselves, was used and composed of sociodemographic data (sex, age, self-reported race, educational level, marital status, income, religion, occupation), clinical data (weight, height, BP, heart rate and pulse); intermittent claudication, comorbidities (diabetes, obesity, dyslipidemia, myocardial infarction-MI, stroke, CHF, and lifestyle habits (sedentary lifestyle, smoking, alcohol consumption) and medications in use (anticoagulant agents, antiplatelet agents, angiotensin-converting enzyme inhibitors [ACEI], angiotensin receptor blockers [ARB], calcium channel blockers-BBC, beta-blocker-BB, statin, nitrate and diuretic).

To measure weight, a platform-type, electronic digital scale (Omron HBF-214), with 150kg capacity and 50g precision was used. Height was measured with the aid of an inextensible measuring tape attached to a wall, with participants standing with their backs to the tape. Body mass index (BMI) was calculated using the ratio weight (Kg)/height^2^(m), and BMI values were considered as normal (BMI between 18.5 and 24.9 Kg/m^2^), overweight (BMI between 25 and 29.9 Kg/m^2^) and obesity (BMI ≥30 Kg/m^2^).^14^

To measure BP, automatic portable devices (model HEM-7113 Omron) and cuffs suitable for upper arm circumference were used. BP measurements were taken in accordance with the Brazilian Arterial Hypertension Guidelines,^1^ with the participant seated and the cuff positioned at heart level, palm facing upward. The participant’s legs were uncrossed, bladder empty, and they had refrained from physical exercise for at least 60 minutes, as well as from consuming alcohol, coffee, food, or smoking in the 30 minutes prior to the measurement. All participants underwent three BP measurements, with a one-minute interval between them, and the average of the last two BP measurements was considered for analysis.

Diabetic individuals were defined as the use of hypoglycemic drugs and/or insulin, according to the medical records, prior to hospital admission. Patients with dyslipidemia were defined as those who presented changes in at least one of the components (HDL-C, LDL-C or triglycerides), or even those who used statins. Patients who smoked at least one cigarette per day were considered smokers. Those who did not perform any physical activity for at least 10 minutes continuously during the week were considered sedentary.

Regarding the consumption of alcoholic beverages, all those who reported consuming alcoholic beverages were considered, regardless of type, quantity or frequency.

To assess adherence to drug or non-drug treatment, the Questionnaire of Adherence to the Treatment of Hypertension (QATSAH) was used, as authorized by the author, consisting of 12 questions and with a Cronbach’s alpha coefficient of 0.81. The scale can vary between 60 and 110, and the higher the score, the greater the adherence to treatment (Level 60: They skip antihypertensive medication at least once a week or take a different dose than the one prescribed at least once a week; Level 70: They neglect to take their hypertension medication at the correct time at least once a week and attend scheduled appointments; Level 80: They neglect to take the antihypertensive medication at the prescribed dose at least once a month; they take the medication independently of symptoms, and reduce consumption of salt and fat by a third; Level 90: They neglect to take their medication at the correct time at least once a month; they reduce the intake of salt, fat, and sweets and sugary drinks by half; take the medication at least once a year and eat practically no fat, no sweets and sugary drinks; Level 100: They neglect to take their hypertension medication at least once a year and eat practically no fat, sweets and sugary drinks; Level 110: They do not neglect to take their hypertension medication, and eat practically no salt and follow non-drug treatment routinely.^15^

To assess the influence of spirituality/religiosity, the DUKE scale (P-durel) was used, which is composed of five items that measure three dimensions of religious involvement related to health outcomes: Organizational Religiosity (OR), Non-Organizational Religiosity (NOR) and Intrinsic Religiosity (IR).^16^OR involves participation in religious services and communal activities associated with religion, while NOR refers to private religious practices such as prayer, meditation, and personal acts of devotion. In contrast, IR focuses on the significance and influence of religion in an individual’s everyday life.^16^

Additionally, the Brief Multidimensional Measure of Religiousness/Spirituality (BMMRS-p) was used to assess the influence of spirituality/religiosity on treatment adherence. The instrument assesses the relationship between religiosity, spirituality and health. The BMMRS-p has 38 items and measures 11 dimensions: 1) Daily spiritual experiences; 2) Values/beliefs; 3) Forgiveness; 4) Private religious practices; 5) Religious coping; 6) Religious support; 7) Spiritual religious history; 8) Commitment; 9) OR; 10) Religious preferences and 11) Global self-assessment of spirituality/religiosity. The answer options are arranged on a Likert scale, with some items ranging from one to eight options and others from one to six. Each dimension has a specific scoring system, where a lower score indicates a higher degree of the dimension in question. To facilitate analysis, item scores can be reversed during data entry so that higher scores reflect greater religiosity/spirituality.^17^

### Statistical analysis

The data were organized in an Excel spreadsheet, validated by double typing, and statistical analysis was performed using the Statistical Package for the Social Science (SPSS Windows), version 25.0^TM^. Continuous variables were described using mean ± standard deviation due to normality of data distribution and categorical variables were expressed using absolute and relative frequencies. The Kolmogorov-Smirnov test was used to assess normality of data distribution. Unpaired Student’s t-test was used to verify the influence of sociodemographic and clinical variables, as well as lifestyle habits, on treatment adherence scores.

The simultaneous influence of variables was analyzed using multiple linear regression, where religion- and spirituality-related variables were initially included together in the model. Multicollinearity was assessed using the Variance Inflation Factor (VIF) and collinearity diagnostics, and no significant issues were identified. Based on conceptual relevance and statistical significance, a final model with nine predictor variables was established. The assumptions for parametric tests were verified, and a significance level of 5% (α = 0.05) was adopted for inferential analyses. As presented in Table 4, the statistical power achieved was 98.7%, considering the sample size of 237 participants, with a significance level at 0.05, nine predictor variables, and a coefficient of determination R^2^ = 0.12.

**Table 4:** – Multiple linear regression analysis of sociodemographic and religious/spiritual factors influencing medication and non-medication adherence in hypertensive patients, adjusted model (QATSAH). Uberlândia, Minas Gerais, Brazil, 2021-2022

Variables	Β	Beta	Sig.	95% CI
Sex	-0.47	-0.03	0.63	[-2.39,1.45]
Age	1.89	0.12	0.073	[-0.18,3.96]
Income	1.93	0.12	0.059	[-0.07,3.93]
Education	0.52	0.03	0.63	[-1.63,2.68]
Comorbities	0.15	0.03	0.56	[-0.36,0.66]
Intrinsic Religiosity	0.67	0.24	0.004	[0.22,1.13]
Daily Spiritual Experiences	0.09	0.06	0.46	[-0.16,0.35]
Values and Beliefs	-0.89	-0.18	0.012	[-1.58,-0.20]
Forgiveness	0.66	0.16	0.015	[0.13,1.19]

*QATSAH: Questionnaire of Adherence to the Treatment of Hypertension. Dichotomous variables: sex (male or female – reference category); age (adult or elderly – reference category); income (< 2,500 Brazilian reals and > 2,500 Brazilian reals – reference category); education (elementary school and some secondary school or more – reference category).*

## Results

Two hundred and thirty-seven hypertensive patients with a mean (±SD) age of 63.0±12.1 years were included. Most patients were male (127; 53.6%), aged ≥60 years (156; 65.8%), catholic (134; 56.5%), had ≤ eight years of formal education (166; 70%), retired (133; 56.1%), and overweight (156; 67.8%); 44.7% (n=106) were white, and 46.8% (n=111) of patients had high level (90th percentile) of adherence to hypertension treatment. Table 1 shows the sociodemographic and clinical profiles, lifestyle habits, medications used, and adherence levels according to the QATSAH questionnaire among hypertensive participants.

**Table 1:** – Sociodemographic and clinical profiles, lifestyle habits, medications used, and adherence levels according to the QATSAH questionnaire among hypertensive participants (n=237). Uberlândia, Minas Gerais, Brazil, 2021-2022

Variables	Value
Male, n (%)	127 (53.6%)
Age, years	63.03±12.06
≥65 years, n (%)	156 (65.9%)
White, n (%)	106 (44.8%)
Formal education	227 (95.8%)
Married, n (%)	104 (41.2%)
Income<1MW, n (%)	145(63.4%)
Religion	
• Catholic, n (%)	134 (56.4%)
• Evangelical, n (%)	57 (24%)
• Spiritist, n (%)	17 (7.2%)
• No religion, n (%)	23 (9.7%)
• Does not believe in God, n (%)	1 (0.4%)
• Candomblé, n (%)	5 (2.1%)
Retired, n (%)	133 (56.2%)
BMI, Kg/m^2^	28.29±6.27
SBP, mmHg	128.86±21.91
DBP, mmHg	75.91±13.09
Heart rate, n (%)	76.90±14.69
Regular pulse, n (%)	211 (88.9%)
Intermittent claudication	58 (24.5%)
DM, n (%)	78 (32.8%)
Obesity, n (%)	42 (17.7%)
Dyslipidemia, n (%)	91 (38.4%)
MI, n (%)	57 (24%)
Stroke, n (%)	29 (12.2%)
ICC, n (%)	49 (20.6%)
Sedentarism, n (%)	154 (65.1%)
Smoking, n (%)	32 (13.6%)
Alcohol consumption, n (%)	36 (15%)
Medication use	
• Anticoagulant, n (%)	16 (6.8%)
• Antiplatelet, n (%)	25 (10.7%)
• ACE inhibitors, n (%)	29 (12.3%)
• ARB, n (%)	144 (61.3%)
• CCB, n (%)	44 (18.7%)
• BB, n (%)	88 (37.6%)
• Statin, n (%)	75 (31.9%)
• Nitrate, n (%)	8 (3.5%)
• Diuretic, n (%)	116 (49.4%)
QATSAH	
• Level 60	1 (0.42%)
• Level 70	11 (4.6%)
• Level 80	80 (33.7%)
• Level 90	111 (46.8%)
• Level 100	31 (13.0%)
• Level 110	3 (1.26%)

*Values are expressed as absolute (n) and relative (%) frequencies or mean and Standard Deviation (SD). MW: minimum wage; BMI: body mass index; SBP: systolic blood pressure; DBP: diastolic blood pressure; DM: Diabetes mellitus; MI: myocardial infarction; CHF: congestive heart failure; ACE inhibitors: angiotensin-converting enzyme inhibitors; ARB: angiotensin receptor blockers; CCB: calcium channel blockers; BB: beta blocker; QATSAH: Questionnaire of Adherence to the Treatment of Hypertension.*

Higher levels of adherence to treatment were observed when aged ≥65 years, non-sedentary, and who did not drink alcohol (p<0.05) (Table 2).

**Table 2:** – Comparison between sociodemographic, clinical variables, lifestyle habits and QATSAH average. Uberlândia, Minas Gerais, Brazil, 2021-2022

	Mean - QATHAS	p
Female	91.8 ±8.3	0.620
Male	92.3 ±7.2	
Age ≥65 years	93 ±7.9	0.009*
Age <65 years	90.4 ±6.9	
White	91.8 ±7.6	0.567
Mixed (Brown)/Black/Asian	92.4 ±7.8	
No formal education	88.3 ±5.3	0.444
Formal education	92.3 ±7.7	
<1 MW	91.9 ±7.9	0.476
≥1 MW	92.6 ±7.4	
Not sedentary	94.9 ±7.6	0.000*
Sedentary	90.6 ±7.3	
Smoker	91.6 ±6.7	0.689
Not Smoker	92.7 ±7.8	
No alcohol consumption	92.5 ±7.9	0.031*
Alcohol consumption	90 ±5.8	

*MW: minimum wage; QATSAH: Questionnaire of Adherence to the Treatment of Hypertension. Student’s t test.*

A multiple linear regression analysis was performed to assess the influence of religious and spiritual factors on adherence (Table 3). Intrinsic religiosity, values and beliefs, and forgiveness were significantly associated with higher QATSAH results. Specifically, higher scores in intrinsic religiosity and forgiveness correlated with greater adherence to hypertension treatment, while lower scores in values and beliefs were linked to better adherence (Central Figure).

**Table 3:** – Multiple linear regression analysis of religious and spiritual factors influencing medication and non-medication adherence among hypertensive patients, based on the Questionnaire of Adherence to the Treatment of Hypertension (QATSAH); Uberlândia, Minas Gerais, Brazil, 2021-2022

Religious and Spiritual Variables	Β	Beta	Sig.	95% CI
Organizational Religiosity	0.364	0.081	0.308	[-0.34,1.07]
Non-Organizational Religiosity	0.158	0.026	0.735	[-0.76,1,08]
Intrinsic Religiosity	0.586	0.208	0.023	[0.08,1,09]
Daily spiritual experiences	0.122	0.081	0.380	[-0.15,0.39]
Values/beliefs	-1.061	-0.219	0.002	[-1.74,-0.38]
Forgiveness	0.696	0.170	0.012	[0.16,1.23]
Religious coping	-0.187	-0.088	0.283	[-0.53,1.04]
Commitment	-0.042	-0.041	0.613	[-0.20,0.12]
Global self-assessment of spirituality/religiosity	0.367	0.083	0.287	[-0.30,0.15]

Based on this broader analysis, four key variables — intrinsic religiosity, daily spiritual experiences, values and beliefs, and forgiveness — were selected for further adjustment due to their referential relevance and statistical significance. These variables were then adjusted for sociodemographic factors (sex, age, income, education, and comorbidities), as shown in Table 4. In the adjusted model, intrinsic religiosity, values and beliefs, and forgiveness remained significant, further reinforcing their influence on adherence.

## Discussion

Aging is often associated with a higher incidence of hypertension, a condition that requires pharmacological interventions to control BP. In many cases, elderly people tend to be more aware of the importance of health care, which includes regular adherence to prescribed medication. In this study, it was observed that those aged 65 years or older had higher levels of adherence to treatment. This fact can be justified by the awareness of the importance of treatment to prevent complications, regular attendance at medical appointments, as well as stability in the daily routine, with more regular times for meals and medications observed in the elderly population.^18,19^

The positive relationship between being physically active and adherence to treatment in hypertensive patients stands out as an important element in maintaining cardiovascular health. In addition to the positive association between BP control and regular physical activity, it is known that physical activity can reduce values of BP within 24 hours after its practice.^20^ Furthermore, individuals who incorporate physical activity into their daily routine tend to adhere to medication prescriptions as well as other guidelines related to a healthy lifestyle, such as maintaining body weight, controlling stress and improving sleep. These data corroborate the findings of this study, in which non-sedentary participants showed higher levels of adherence to treatment.

There is a consensus among research studies that excessive alcohol consumption can negatively influence BP, increasing the risks of hypertension and compromising the effectiveness of antihypertensive medications, as well as constituting an important barrier to treatment adherence. In this study, it was observed that participants who did not consume alcohol showed higher levels of adherence to treatment. Individuals who choose not to consume alcohol generally demonstrate greater commitment to medical recommendations, which may include taking prescribed medications regularly. Furthermore, not consuming alcohol reduces the likelihood of unwanted drug interactions, which favors the effectiveness of pharmacological treatment, resulting in adherence to treatment. Also, not consuming alcohol contributes to maintaining an adequate body weight, reducing the risk of obesity, which can improve the clinical picture of hypertension.^21,22^

Religiosity and spirituality have been identified in numerous studies as factors that promote adherence to medication treatment. For example, one study found that individuals with higher levels of religiosity are more likely to adopt healthier lifestyles, which may contribute to better treatment adherence.^23^ Furthermore, research indicates that patients who engage in practices such as prayer and meditation, or seek support from their religious communities tend to experience fewer symptoms of depression, which can further facilitate adherence to treatment.^23^ Various mechanisms may explain this phenomenon. Religiosity and spirituality can foster a sense of hope and purpose, which enhances an individual’s commitment to treatment. Additionally, community involvement and practices such as avoiding some foods and harmful substances, often encouraged by religious groups, may also play a key role in promoting adherence.^23,24^

Intrinsic religiosity refers to the deep and personal experience of faith, where spirituality plays a significant role in the individual’s life. The data listed here indicate that the greater the intrinsic religiosity of a hypertensive patient, the greater the adherence to treatment. Likewise, a study carried out with 9,581 adult Adventist men and women found that intrinsic religiosity is a significant factor in reducing hypertension rates.^25^ The reason for this association is not yet clear, but we can think that individuals with high intrinsic religiosity find in their faith a source of emotional support, inner strength and meaning to face challenges, including health management. Thus, the belief in a greater purpose can motivate the adoption of healthy behaviors and adherence to medical recommendations, thus contributing to better control of hypertension. A high intrinsic religiosity can be a valuable resource in promoting adherence to hypertension treatment, providing emotional support and a sense of purpose and positively influencing healthy lifestyle habits. However, it is essential to consider the individuality of each person when exploring this interconnection between spirituality and health.^26^

In this study, forgiveness was also related to greater adherence to treatment. Forgiveness is understood as the ability to let go of resentment and hostility towards others. Individuals who cultivate a willingness to forgive often experience reduced levels of stress, anxiety and anger, which, when present in excess, can contribute to the development and worsening of hypertension.^27^ In a study carried out with 3,105 hypertensive patients, it was found that the forgiveness variable was associated with lower diastolic BP and a reduced probability of hypertensive outcomes.^28^ Individuals undergoing a forgiveness training program had significant reductions in their BP when compared to those who did not participate in the program.^29^ Therefore, we can infer that the ability to forgive is associated with an improvement in psychological health, promoting a state of well-being that can be positively reflected in lifestyle choices and adherence to medical recommendations.

Forgiveness is also related to promoting healthy behaviors. Individuals who cultivate the ability to forgive may be more likely to take a proactive approach to their health, including consistent adherence to medication, balanced eating habits, and engaging in regular physical activity. These elements are fundamental in the effective control of hypertension. Furthermore, the practice of forgiveness is associated with an improvement in interpersonal and social relationships. Healthier family and community relationships can create a supportive environment where adherence to treatment is encouraged and facilitated. Social support is recognized as a crucial factor in the effective management of hypertension and the promotion of healthy behaviors.^28,30^

However, in relation to values and beliefs, a negative influence on adherence to treatment was found. Values and beliefs refer to fundamental convictions about the way people interpret and interact with the world, that is, they are intrinsic elements of individuals’ identity, shaping their perceptions about health, treatment and lifestyle. Therefore, when a person’s values and beliefs conflict with medical recommendations for controlling hypertension, adherence to treatment may be compromised. Cultural, spiritual, and philosophical beliefs can play a significant role in how a person addresses hypertension treatment. Also, a distrust regarding the effectiveness of conventional treatments, often rooted in personal beliefs, can lead to the search for alternative approaches or hesitation in following medical advice.^31^

Thus, identifying and supporting religious coping and forgiveness strategies, as well as understanding the values and beliefs of hypertensive patients, can promote and facilitate greater adherence to treatment. It is crucial that healthcare professionals play an active role in this process, not only recognizing and validating the importance of spirituality and religious practices for their patients, but also integrating this understanding into personalized care strategies. By doing so, they can identify possible barriers to treatment adherence based on beliefs and work collaboratively to find solutions that respect these views, while encouraging effective health practices.^32^

### Study limitations

Due to the cross-sectional design, limited sample size, and the fact that the study was conducted in a single hospital, causal inferences and extrapolation of the results to other contexts should be made with caution. Moreover, the cross-sectional nature of the study limits the ability to establish cause-and-effect relationships between factors like spirituality/religiosity and treatment adherence in hypertensive patients. Additionally, most data were self-reported, which introduces the potential for recall bias. Adherence was assessed indirectly using patient self-reports through the QATSAH questionnaire.

## Conclusion

Religiosity/spirituality influenced medication and non-medication adherence in hypertensive individuals, in the dimensions Intrinsic Religiosity, Values and Beliefs and Forgiveness. These findings suggest that investigations and interventions focused on psychosocial and behavioral elements are essential to improve the population’s health outcomes in terms of controlling/maintaining hypertension.
